# Uneven effects of trans-vaginal mesh reconstruction on the viscoelastic property of the urinary bladder in patients with pelvic organ prolapse

**DOI:** 10.3389/fbioe.2025.1677779

**Published:** 2026-01-12

**Authors:** Hui-Hsuan Lau, Cheng-Yuan Lai, Ming-Chun Hsieh, Hsien-Yu Peng, Dylan Chou, Tsung-Hsien Su, Jie-Jen Lee, Tzer-Bin Lin

**Affiliations:** 1 Division of Urogynecology, Department of Obstetrics and Gynecology, Mackay Memorial Hospital, Taipei, Taiwan; 2 Department of Medicine, Mackay Medical University, New Taipei, Taiwan; 3 Institute of Biomedical Sciences, Mackay Medical University, New Taipei, Taiwan; 4 Department of Surgery, Mackay Memorial Hospital, Taipei, Taiwan; 5 Institute of Translational Medicine and New Drug Development, College of Medicine, China Medical University, Taichung, Taiwan; 6 Department of Physiology, School of Medicine, College of Medicine, Taipei Medical University, Taipei, Taiwan; 7 Graduate Institute of Biomedical Electronics and Bioinformatics, National Taiwan University, Taipei, Taiwan

**Keywords:** dynamic compliance, pelvic reconstruction, transvaginal mesh repair, viscoelasticity, volume-pressure analysis

## Abstract

**Introduction:**

The pathophysiology underlying deficits in bladder storage and possible rationale for how transvaginal mesh (TVM) reconstruction benefits bladder function in patients with pelvic organ prolapse (POP) remain unclear. Compliance, a viscoelastic parameter, crucially characterizes the bladder’s storage function. Pressure-volume analysis (PVA) has been recently applied to specifically assess compliance dynamics during different stages of bladder filling. This study investigated compliance dynamics in patients with POP using PVA and analyzed the impact of TVM on bladder compliance.

**Methods:**

PVAs were retrospectively constructed by plotting intravesical volume (Vive) against detrusor pressure (Pdet) in patients with POP undergoing TVM repair. Parameters analyzed included mean compliance during the entire, early, and late halves of bladder filling (Cm, C1/2, and C2/2, respectively); infused volume (Vinf); threshold pressure (Pthd); Pdet changes in the early and late filling (ΔPthd1/2 and ΔPthd2/2, respectively); post-void residual volume (Vres); and ultrasound imaging.

**Results:**

Before TVM, patients exhibited significantly lower C2/2 than C1/2 (p < 0.001, N = 22), while postoperatively, no significant difference was observed between C2/2 and C1/2 (p = 0.102, N = 22). TVM significantly increased Cm (p = 0.004, N = 22) and C2/2 (p < 0.001, N = 22) but had no significant effect on C1/2 (p = 0.572, N = 22). Postoperatively, Pthd (p = 0.001, N = 22) and ΔPthd2/2 (p = 0.001, N = 22) were significantly reduced, while ΔPthd1/2 (p = 0.084, N = 22) and Vinf (p = 0.112, N = 22) remained unaffected. Ultrasound imaging demonstrated that TVM relieved restrictions on bladder expansion during late filling.

**Discussion:**

Patients with POP showed heterogeneous compliance deficits, particularly during late bladder filling. TVM selectively improved compliance impairment possibly by restoring anatomical geometry for bladder expansion.

**Clinical Trial Registration:**

ClinicalTrials.gov (NCT05682989).

## 1 Introduction

The urinary bladder, as a highly compliant organ, exhibits only slight increases in pressure despite significant volume expansion during urine storage (Sugaya et al., 2000). As compliance is a critical viscoelastic parameter defining bladder storage function, compromised compliance can abnormally elevate bladder pressure, potentially damaging the upper urinary tract (Wu and Franco, 2017) or causing urinary incontinence (Arunachalam and Heit, 2020).

Among aging women, the prevalence of pelvic organ prolapse (POP), defined as the herniation of pelvic organs beyond their anatomical boundaries, is rising due to increasing life expectancy. POP predominantly affects women over 70 years of age (Iglesia and Smithling, 2017; Aichner et al., 2022). Notably, women with advanced POP exhibit lower bladder compliance during urgency compared to healthy controls (Long et al., 2002). Urodynamic studies reveal that bladder compliance in patients with POP is significantly lower during late filling stage compared to early one (Lau et al., 2024c), suggesting that POP is associated with reduced bladder compliance, particularly during late bladder filling.

Transvaginal mesh (TVM) reconstruction, which addressed anatomical abnormalities in the pelvic cavity (Majkusiak et al., 2015), is a minimally surgical option for POP repair (Luo et al., 2018). Despite safety concerns raised by the U.S. FDA regarding the long-term use of TVM (US Food and Drug Administration, 2016), it remains a viable and commonly used option in Asia and Continence Europe (Rogowski et al., 2019; Naumann et al., 2021; Mateu-Arrom et al., 2021), where it has demonstrated satisfactory anatomical and functional outcomes (Long et al., 2012). Importantly, beyond structural correction and symptom relief, there is a critical need to objectively assess how TVM affects bladder functions, particularly bladder storage-as restoring function is a key goal of pelvic floor surgery.

In clinical practice, time-domain cystometry is widely used to measure static bladder compliance, typically calculated offline by dividing the volume change by the pressure change over the filling phase (Cameron et al., 2016). While informative for mean compliance, this method is limited in evaluating specific phase of the bladder filling cycle-such as the initial filling stage or maximum capacity.

Pressure-volume analysis (PVA), derived from cystometry (Lau et al., 2022; Lau et al., 2023; Lau et al., 2024b), has been shown to effectively illustrate compliance dynamics across voiding cycles. PVA has been used not only in animal studies under physiological (Peng et al., 2020) and pharmacological (Peng et al., 2021) conditions but also to assess the therapeutic effects of conservative pelvic floor therapy (Lau et al., 2024a) and surgical interventions (Lau et al., 2024c) in human patients.

Given prior evidence that POP-related compliance deficits are most pronounced during late bladder filling (Long et al., 2002; Lau et al., 2024c), we hypothesized that compliance impairments in POP are heterogeneous across the filling cycle, and that TVM may differentially affect these stages. Therefore, in this study we utilized PVA to evaluate compliance during the early, late, and entire bladder filling phase in patients with POP undergoing TVM reconstruction.

## 2 Patients and methods

### 2.1 Patient database

In accordance with the Declaration of Helsinki, this study was approved by the Institutional Review Board (Ethics Committee of Mackay Memorial Hospital, Taipei, Taiwan; Approval number: 22MMHIS361e; Date: 2022/12/08) and registered on ClinicalTrials.gov (Registration number: NCT05682989). Medical records of patients from January 1, 2007 to December 31, 2023 at university hospitals were retrospectively analyzed. The inclusion criteria was female patient underwent primary TVM repair for symptomatic POP with a grade higher/equal to stage II (POP quantification system). Patients who failed to complete pre- and post-operative follow-ups, had a history of vesico-vaginal, recto-vaginal, or urethra-vaginal fistula, or under radiation therapy were excluded from this study.

### 2.2 Surgery

A midline incision was made on the anterior vaginal wall, extending from the level of the bladder neck to the cervix. After hydro-dissection, a full-thickness vesico-vaginal plane was identified. A modified mesh (Surelift®, Neomedic International, Barcelona, Spain) was used, in which the two central arms were trimmed. The posterior arms were anchored to the sacrospinous ligaments. Using circular needle passers, the two anterior arms were passed through the obturator foramina. The mesh was adjusted to ensure it was tension-free without folding. To prevent mesh migration, sutures were placed below the bladder neck near the vaginal vault. Finally, the vaginal epithelium was closed.

### 2.3 Cystometry and PVAs

**FIGURE 1:**
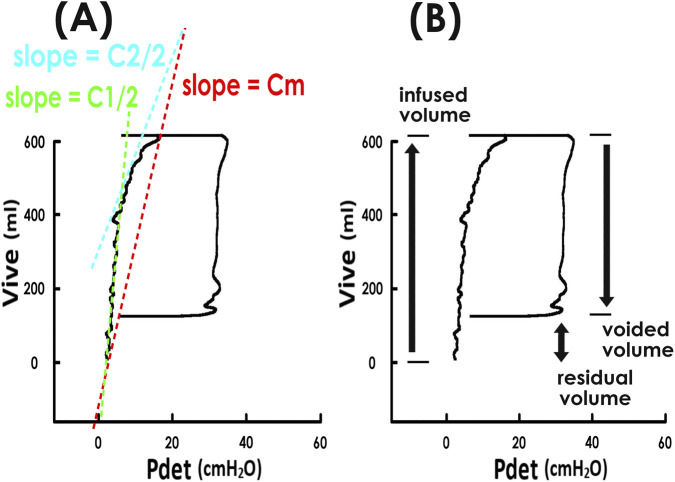
Pressure-volume analyses and associated parameters. **(A)** and **(B)** Pressure-volume analyses of voiding cycles constructed by plotting intravesical volume (Vive) against detrusor pressure (Pdet). The trajectory forms a loop; the left border represents bladder filling. **(A)** The slopes of the tangent lines in the entire, early half, and late half of bladder filling (red, green, and blue dashed lines, respectively) represent mean compliance at each stage-Cm, C1/2, and C2/2. **(B)** Post-void residual volume (double arrow) is calculated as the difference between infused volume (upward arrow) and voided volume (downward arrow).

In our hospitals, urodynamic investigations were routinely performed before and after an invasive urogynecological surgery to evaluated bladder function in detail. During the cystometry, vesical, abdominal, and detrusor pressures (Pves, Pabd, and Pdet, respectively), urethral flow (Flow), and infused, voided, and intravesical volumes (Vinf, Vvod, and Vive, respectively) were recorded. PVAs were constructed by plotting Vive against Pdet (Figures 1A,B) (Peng et al., 2021; Lau et al., 2024a). Bladder compliances for the entire filling phase (Cm, red), early half (C1/2, green) and late half (C2/2, blue) were calculated as the slope of the tangent lines in the corresponding segments (Figure 1A). Post-void residual volume (Vres) was calculated as the difference between Vinf and Vvod (Figure 1B).

### 2.4 Ultrasound images

Transabdominal ultrasound examinations were performed with the patient in a dorsal lithotomy position. An ultrasound system (Voluson S6/8, GE Healthcare, Zipf, Austria) equipped with a transabdominal transducer (4C-RS/C1-5-RS; GE Healthcare), operating at a frequency of 1.5–5 MHz, gain settings from −5 to +5, and a volume acquisition angle of 60° was used to assess the urinary bladder. Sagittal and transverse images were captured at the end of the early and late halve of bladder filling for analysis. Bladder volume was estimated by ellipsoid formula, i.e., volume = 0.52 × width × length × height. The assessor was kept blind to pre- or post-operative status of patients.

### 2.5 Statistical analysis

Baseline characteristics of patients (age and intervals between the surgery and pre-/post-operative evaluations) were presented using descriptive statistics. Quantitative data were presented as mean ± S.E.M. Wilcoxon signed rank test were used to compare pre- and post-treatment values. P-values, which were initially set less than 0.05 as statistically significant, was adjusted by Benjamini hochberg correction (supplementary data). No missing data was observed during analysis.

## 3 Results

### 3.1 Patient database

Cystometry was analyzed from 22 female patients (mean age = 67.22 ± 5.78 years; mean parity = 3.04 ± 0.25). Cystometric evaluations were conducted at a mean of 39.72 ± 43.36 days before and 112.22 ± 25.27 days after TVM surgery. All patients had POP ≥ stage II according to the POP quantification system (15 patients were stage III and 7 patients were stage IV). Three patients had a history of prior pelvic surgery (for ectopic pregnancy, Cesarean section, and anal fistula). Comorbid medical conditions included hypertension (16 patients), hyperlipidemia (8 patients), diabetes (7 patients), arrhythmia (1 patient), and coronary artery disease (1 patient). No patients had neurogenic bladder or used medications that might affect bladder function.

### 3.2 Graphical assessment of bladder compliance

**FIGURE 2:**
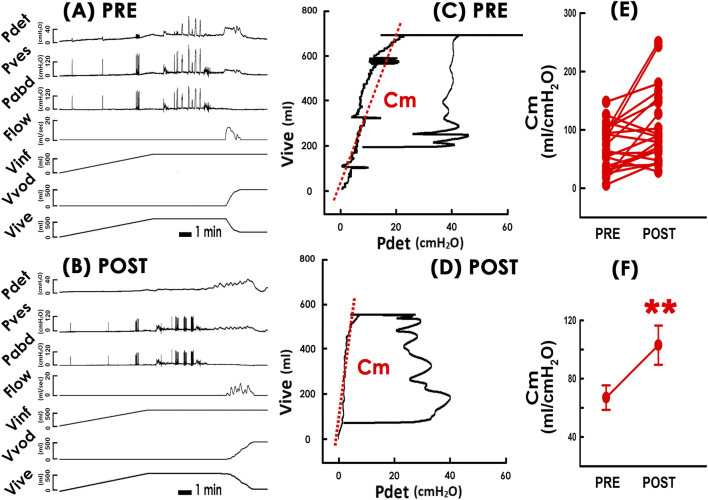
Impacts of TVM on the bladder compliance. **(A)** PRE and **(B)** POST Representative cystometry data before and after TVM, respectively. **(C)** PRE and **(D)** POST Pressure-volume analyses before and after TVM. Slopes of red dashed lines indicate the mean compliance (Cm). Note: Loops show more infused than voided volume, indicating residual volumes of urine. **(E)** Individual and **(F)** mean Cm values measured pre- and post-TVM (PRE and POST, respectively). **p = 0.004, power = 0.866, vs. PRE; N = 22. Pdet: detrusor pressure, Pves: vesical pressure, Pabd: abdominal pressure, Flow: urethral flow, Vinf: infused volume, Vvod: voided volume, Vive: intravesical volume.

PVAs were constructed from pre- and post-operative cystometry data (Figure 2A PRE and Figure 2B POST) by plotting intravesical volume (Vive) against detrusor pressure (Pdet) (Figures 2C,D). In these PVAs, the trajectory formed a closed loop representing a voiding cycle. The left border of the loop, where Vive gradually elevated with slight Pdet increase, represented the bladder filling phase. The slope of the tangent line along the left border (red dashed lines) was calculated to determine the mean compliance (Cm) during bladder filling.

### 3.3 TVM increases bladder compliance

Compared to the pre-operative state (Figure 2C PRE), representative PVAs showed that TVM distinctively increased mean compliance (Cm), indicated by a counterclockwise tilt of the tangent line (Figure 2D POST). Statistical analysis confirmed this increase as TVM increased Cm in the majority of patients consistently (Figure 2E; 17 out of 22 (17/22; 77%)) and significantly increased the group mean Cm (Figure 2F. p = 0.004, power = 0.866; N = 22).

### 3.4 TVM increases compliance during late bladder filling

Given that patients with POP often exhibits storage dysfunction, particularly under urgency (Long et al., 2002; Lau et al., 2024c), we wondered whether bladder compliance was impaired heterogeneously during filling and whether TVM affected compliance unequally across filling stages. To clarify this question, we measured compliance during the early and late halves of bladder filling (C1/2 and C2/2, respectively), and assessed the effects of TVM on both.

**FIGURE 3:**
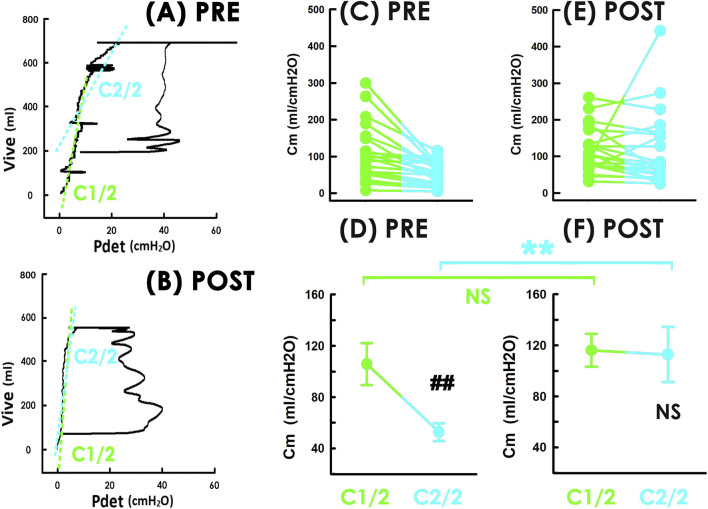
Impacts of TVM on compliance during early and late bladder filling. **(A)** PRE and **(B)** POST Pressure-volume loops before and after TVM for a representative patient. Slopes of green and blue dashed lines indicate compliance of early and late stage of filling. **(C)** individual and **(D)** mean C1/2 (green) and C2/2 (blue) before TVM. ##p < 0.001, power= 0.974, vs. C1/2 **(E)** individual and **(F)** mean C1/2 and C2/2 after TVM. NS p = 0.102, power = 0.053, vs. C1/2; NS p = 0.572, power = 0.085, vs. PRE (green); **p < 0.001, power = 0.896, vs. PRE (blue); all N = 22. Vive, intravesical volume; Pdet, detrusor pressure.

Preoperative PVAs showed that during late filling, the trajectory shifted rightward and downward (Figure 3A PRE), causing the tangent line for the late phase (blue dashed line) to tilt clockwise compared to the early phase (green dashed line) indicating lower C2/2 than C1/2. Whereas, in postoperative PVA, the trajectory remained stable without such turning (Figure 3B POST, and the tangent lines for early and late filling (green and blue dashed lines, respectively) displayed similar slopes, suggesting C1/2 and C2/2 were comparable.

Summarized data revealed that prior to TVM, nearly all patients consistently showed lower C2/2 than C1/2 (Figure 3C; 21/22 (95%)), and the mean C2/2 was significantly lower (Figure 3D PRE, p<0.001, power = 0.974; N = 22). After TVM, although 17 patients still had lower C2/2 than C1/2 (Figure 3E), the group mean difference was no longer significant (Figure 3F, p = 0.102, power = 0.053; N = 22).

Moreover, TVM did not consistently affect individual C1/2 (14/22 (64%) increased and 8/22 (36%) decreased) or significantly change the mean C1/2 (Figures 3D,F; p = 0.572, power = 0.085; N = 22). However, it consistently increased C2/2 in most patients (17/22 (77%)) and significantly increased mean C2/2 (Figures 3D,F; p<0.001, power = 0.0896 N = 22). These findings indicate that compliance impairment in patients with POP is heterogeneous and that TVM selectively improved compliance during the late filling phase.

### 3.5 TVM relieves limitations in bladder expansion

**FIGURE 4:**
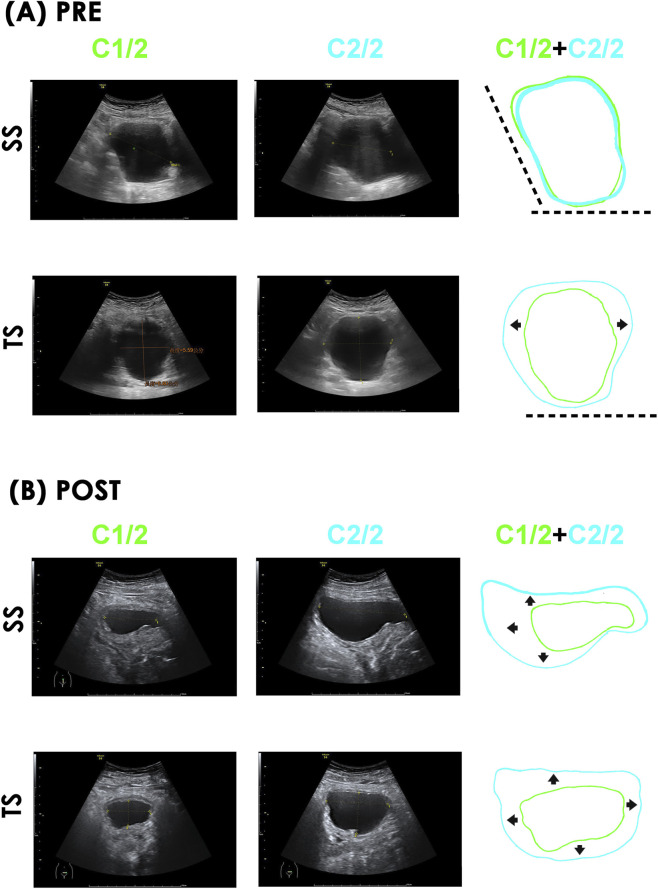
Impacts of TVM on bladder distension during late filling. **(A)** PRE and **(B)** POST Pre- and post-operative ultrasound images of a representative patient. Sagittal and transverse section (SS and TS, respectively) captured at the ends of early (C1/2, green) and late (C2/2, blue) bladder filling. Superimposed diagrams (C1/2+C2/2) also shown. **(A)** SS, bladder expansion was restricted rostrally (left dashed line) and dorsally (bottom dashed line). TS, bladder expansion was dorsally limited (bottom dashed line) with bilateral expansion (left/right arrows). **(B)** SS, bladder postoperatively expansion rostrally (left arrow), ventrally (up arrow), and dorsally (down arrow). TS, bladder postoperative expansion occurred ventrally (up arrow), dorsally (down arrow) and bilaterally (left and right arrows) without marked restriction.

Having observed selective improvement in late-phase compliance, we further examined TVM’s effect on bladder geometry using pre- and post-operative ultrasound images captured during early and late filling. Pre-operative images showed limited bladder expansion during the transition from early to late filling (Figure 4A C1/2 and C2/2, respectively), as sagittal sections (SS) lacked noticeable rostral and dorsal distension (left and bottom dashed lines, respectively), and transverse sections (TS) showed no evident dorsal expansion (bottom dashed lines). The bladder primarily distended bilaterally (left and right arrows) during late filling. The preoperative bladder volumes of C1/2 and C2/2 were 131.8 and 235.8 mL, respectively. In contrast, postoperative images of this patient demonstrated adequate bladder expansion, i.e., sagittal sections showed rostral, ventral, and dorsal distension (Figure 4B; left, upward, and downward arrows, respectively) and transverse sections revealed dorsal, ventral, and bilateral distension (Figure 4B; downward, upward, and bilateral arrows, respectively) during filling. These results indicate that TVM relieved mechanical limitations on bladder expansion during late filling. The postoperative bladder volumes of C1/2 and C2/2 pwere 34.6 and 141.7 mL, respectively.

### 3.6 TVM reduces bladder pressure

**FIGURE 5:**
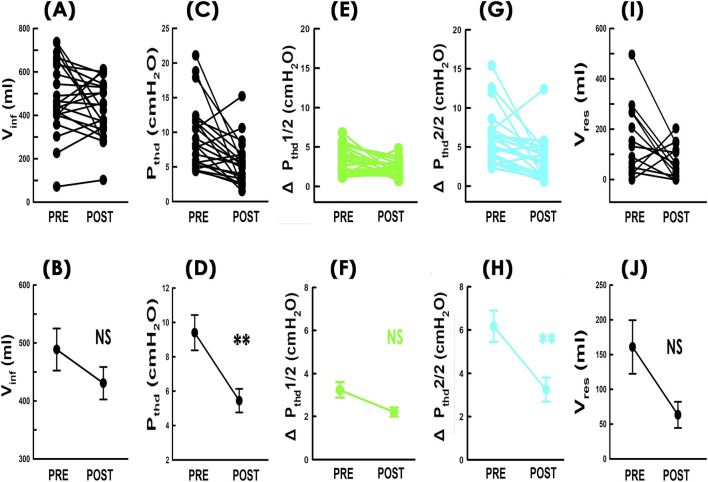
Effects of TVM on compliance-associated parameters. Individual (upper) and mean (lower) values of **(A,B)** infused volume (Vinf; NS p = 0.112, power = 0.353, N = 22), **(C,D)** threshold pressure (Pthd; **p = 0.001, power = 0.878, N = 22), **(E,F)** detrusor pressure change during early filling (ΔPthd1/2; green; NS p = 0.084, power = 0.409, N = 22). **(G,H)** detrusor pressure change during late filling (ΔPthd2/2; blue; **p = 0.001, power = 0.931, N = 22), and **(I,J)** post-void residual volume (Vres; NS p = 0.033, power = 0.600, N = 14) before and after TVM. all vs. PRE.

To investigate possible mechanisms behind increased compliance, we analyzed infused volume (Vinf) and threshold pressure (Pthd) considering that Cm=Vinf/Pthd. TVM did not cause consistent changes in Vinf (Figure 5A; 8/22 (36%) increased and 14/22 (64%) decreased) or significantly altered the mean Vinf (Figure 5B; p = 0.112, power = 0.353; N = 22). However, it consistently reduced Pthd in most patients (Figure 5C; 18/22 (82%)) and significantly lowered the mean Pthd (Figure 5D; p = 0.001, power = 0.878; N = 22), suggesting that the compliance increase was primarily due to reduced bladder pressure rather than changes in infused volume.

We then analyzed changes in detrusor pressure during early and late filling (ΔPthd1/2 and ΔPthd2/2, respectively). TVM did not consistently affect ΔPthd1/2 (Figure 5E; 7/22 (32%) increased and 15/22 (68%) decreased), and the change in mean ΔPthd1/2 was not statistically significant (Figure 5F; p = 0.084, power = 0.409; N = 22). In contrast, ΔPthd2/2 decreased consistently in most patients (Figure 5G; 19/22 (86%)) and showed a significant reduction in mean ΔPthd2/2 (Figure 5H; p = 0.001, power = 0.931; N = 22), indicating that TVM selectively reduced pressure during late filling.

### 3.7 TVM decreases residual volume

Finally, we investigated whether the increase in compliance could be due to postoperative urine retention because past reports have shown that lasting urine retention could result in increased bladder compliance (Yokoyama et al., 1997).

Preoperative PVAs in 14 out of 22 patients showed more infused than voided volume, indicating post-void residual volume (Vres) (Figure 2C PRE). After surgery, although the difference between the top and bottom borders of the loop remained similar, the loop shifted downward, suggesting reduced Vres (Figure 2D POST). Statistical analyses demonstrated that TVM consistently decreased Vres in individual patients (Figure 5I; 11/14, 79%). Although the mean Vres value was reduced, the change did not reach statistical significance after correction. (Figure 5J; p = 0.033, power = 0.600; N = 14). For the remaining 8 patients who emptied their bladder completely before the surgery, no postoperative Vres was detected. These results indicate that TVM cause urinary retention to increase was less likely.

## 4 Discussion

### 4.1 TVM increased bladder compliance

In Continental Europe and Asia, TVM remains a surgical option for POP repair because it achieves satisfactory anatomical reconstruction and objectively relieves both symptoms and signs in patients (Naumann et al., 2021), despite concerns raised by the U.S. Food and Drug Administration about its long-term safety (US Food and Drug Administration, 2016). Though patients with POP are more likely to have emptying problem, and studies have consistently demonstrated the benefits of TVM on voiding functions (Loo et al., 2020; Ma et al., 2021; Lau et al., 2023). Considering intact bladder function involves adequate storage and sufficient emptying, the current study conversely explored the potential advantages of TVM on bladder storage with a focus on bladder compliance as it is a key viscoelastic parameter characterizing storage function. Our results showed that TVM increased bladder compliance in patients with POP-that is, the bladder exhibited reduced pressure increases in response to accumulating volume during filling. Because abnormally elevated bladder pressure during filling can over-excite voiding reflexes (Mills et al., 2000), cause urine reflux (Wu and Franco, 2017) or lead to stress urinary incontinence (Hsieh et al., 2019), we suggest the TVM-induced increase in compliance clinically benefits patients with POP by avoiding upper urinary tract damage and or urinary incontinence secondary to aberrantly elevated intravesical pressure (Wu and Franco, 2017; Arunachalam and Heit, 2020) because our analyses showed the bladder postoperatively developed lower pressure with a barely affected infusion volume.

Further analysis of compliance during early and late stages of bladder filling revealed that preoperatively, the PVA trajectory turned to rightward and downward during late filling, indicating lower compliance in that phase. This was statistically confirmed as before TVM, C2/2 was consistently and significantly lower than C1/2. Although a universal cut-off value for low bladder compliance yet to be established, previous studies define low compliance as ranging from 12.5–40 cc/cmH2O as low compliance in humans (Arunachalam and Heit, 2020; Toppercer and Tetreault, 1979; Harris et al., 1996). In the current study, the mean preoperative C1/2 was 105.82 ± 76.95 cc/cmH2O-relatively higher than the threshold for low compliance, suggesting the compliance deficit in patients with POP was uneven and more pronounced during late filling. This speculation could be supported by previous findings that patients with POP demonstrate compliance deficits particular under urgency (Long et al., 2002) and during late bladder filling (Lau et al., 2024c).

Postoperatively, the PVA trajectory displayed a relatively constant slope throughout filling without noticeable deviation. Summarized data indicated that C1/2 and C2/2 were statistically indistinguishable. Given that mean postoperative values for Cm (103.01 ± 63.04 cc/cmH2O), C1/2 (116.13 ± 60.62 cc/cmH2O), and C2/2 (112.84 ± 101.16 cc/cmH2O) in the current study were all above the levels typically associated with low compliance, and that no significant difference was found in C1/2 before and after surgery, these findings collectively suggest that TVM improves bladder storage function possibly by selectively correcting compliance deficits that occurs during late filling.

### 4.2 Possible mechanisms involved

The pathophysiological basis of our observation that compliance impairment in patients with POP seems occur mainly during late filling and the rationale behind the uneven effect of TVM is unclear. Notably, instead of a uniformly compliance decrement throughout the filling cycle, patients exhibited lower compliance mostly during late filling, suggesting the impairment was less likely due to a global change in the bladder’s viscoelastic properties, such as generalized connective tissue accumulation or increased bladder wall thickness. Moreover, as noted above, C1/2 values before and after TVM were statistically similar and both well above the threshold for low compliance, suggesting that the bladders’ viscoelastic properties in early filling remained relatively unaffected regardless of surgical intervention.

Considering that abrupt bladder compression elevates intravesical pressure in patients (Berkman et al., 2021), and that a pre-clinical study demonstrated that disrupted bladder geometry reduces compliance in rabbits (Çelebi et al., 2016), we propose that the compliance impairment during the late filling of patients with POP is possibly attributed to the restricted bladder expansion caused by mal-aligned pelvic organs. While the bladder may distend without marked restriction in early filling, the substantial volume increase during late filling pushes it into constrained anatomical spaces, resulting in non-physiological geometric deformation. We suggest such abnormal overstretching possibly shifts the detrusor muscle’s length-tension curve toward a higher tension, which in turn produces higher pressure for a given volume (Habteyes et al., 2017), thus manifesting as decreased compliance.

Our conjecture could be supported by preoperative ultrasound images showing that bladder expansion was limited rostrally and dorsally during late filling, with significant bilateral and minor ventral expansion. Combined with the PVA findings indicating reduced compliance during late filling these findings suggests that mechanical restriction due to organ mis-alignment could primarily account for the heterogeneous compliance deficit. Interestingly, in this study, patients underwent TVM to correct their anatomical abnormalities. By relieving the mechanical restriction on bladder expansion caused by the mal-aligned organ-particularly during late filling-TVM, appears to enhance compliance. This finding could be further supported by postoperative ultrasound images which showed marked improvement in rostral, ventral, and dorsal bladder expansion during late filling-unlike the preoperative condition. Together with PVA showing postoperative increases in C2/2 but insignificant change in C1/2, and urodynamic analyses showing TVM reduced bladder pressure changes in late filling but had minimal effect during early filling. Our findings suggest that TVM selectively relieves compliance impairment by correcting anatomical restrictions that elevate bladder pressure during late filling.

Consistent with previous studies showing patients with POP exhibit impaired compliance particularly in late filling (Lau et al., 2024c), the present study is the first to provide imaging evidence that partially explains the possible pathophysiology and the therapeutic rationale for TVM in improving bladder storage function in patients with POP. However, future studies incorporating real-time ultrasound imaging during compliance assessment (Bu et al., 2020) and direct biomechanical or histological data will be essential to further validate this effect. In addition, as lasting bladder stretch misfiring the mechanical receptor in the urothelium releasing neurotransmitter that trigger bladder contractility (Cameron 2019), whether the observe compliance change involves modifed detrusor tone or changes in sensory signaling needs future studies.

### 4.3 TVM ameliorated voiding dysfunctions

In addition to bowel and/or sexual dysfunction, patients with POP also exhibit voiding dysfunctions (Kummeling et al., 2013; Lau et al., 2024d). The results of this study showed that in patients who exhibited urine retention before surgery, TVM unlikely to increase the amount of post-void residual volume. Moreover, in patients who were able to completely empty their bladder before surgery, TVM did not induce urine retention. Given that prolonged urine retention can lead to recurrent urinary tract infections (Dörflinger and Monga, 2001) or upper urinary tract damage (Wu and Franco, 2017; Lyu et al., 2022), our results suggest that TVM at least did not worsen voiding function in patients with POP postoperatively. Future studies investigating the potential benefit of mesh repair to other pelvic problems such as rectal prolapse (Martini et al., 2023) and inguinal bladder hernia (Martini et al., 2022) are warranted to provide relevant clinical insights into mesh-based reconstruction affecting pelvic and urinary structures.

### 4.4 Strengths and limitations

In conjunction with cystometry, this study investigated the possible pathophysiological basis of compliance deficits and the potneitial impact of TVM on compliance in patients with POP using PVA. Unlike traditional methods that assess mean compliance over the entire filling phase, PVA graphically and conceptually illustrates the volume-pressure relationship, allowing targeted analysis of compliance dynamics in both early and late stages. With advances in computer technology, displaying PVA simultaneously with cystometry is now feasible, and real-time application of PVA during ongoing cystometry can effectively evaluate voiding urodynamics and bladder viscoelasticity, providing valuable insights for both research and clinical practices.

However, as a study of retrospective design that analyzed laboratory data, findings in this study inherently have limitations in both internal and external validity of findings. The sample size of this study is small that may introduce bias in effect estimation and limit the generalizability of results. In addition, lacking of a control group in this study limits the causal inference regarding effects of TVM. Given that the benefit and complication of pelvic reconstruction require long-term monitoring with extended observation period, as the postoperative measurements were taken at an average of 112.22 ± 25.27 days after TVM, the improved compliance observed in this study needs future prospective, multicenter studies with longer follow-up and integration of symptom questionnaires to be further validate.

Moreover, history from 2007 to 2023 of POP patient was retrospectively analyzed, considering procedure variations and device evolutions, the measured outcome could be confounded without standardized surgical technique and peri-operative care; and the heterogeneity of patients could be a potential source of bias. Additionally, potential bias from operator dependent in ultrasound and cystometry interpretation needs to be considered.

Because an ultrasound investigation during pre- and post-operative urodynamic measurement is not a routine in our hospitals, statistical analysis of ultrasound image was lacked. Future studies analyzing ultrasound images with uro-/thermos-dynamic data of bladder is necessary to further elucidate the finding presented in this study.

## 5 Conclusion

This study demonstrated that pathological conditions unevenly affect bladder compliance at stages of filling, and that TVM selectively improve compliance deficits occurring during late filling in patients with POP. These findings provide possible support that TVM benefits patients with POP by preventing the adverse effects of abnormally reduced compliance, which can lead to elevated bladder pressure and potential upper urinary tract damage.

## Data Availability

The datasets presented in this study can be found in online repositories. The names of the repository/repositories and accession number(s) can be found in the article/Supplementary Material.

## References

[B1] Aichner S. Fähnle I. Frey J. Krebs J. Christmann-Schmid C. (2022). Impact of sacrocolpopexy for the management of pelvic organ prolapse on voiding dysfunction and roflowmetry parameters: a prospective cohort study. Arch. Gynecol. Obstet. 306 (4), 1373–1380. 10.1007/s00404-021-06369-0 34988660

[B2] Arunachalam D. Heit M. (2020). Low bladder compliance in women: a clinical overview. *Female Pelvic Med. Reconstr. Surg*. . 26 (4), 263–269. 10.1097/SPV.0000000000000666 30520742

[B3] Berkman T. Naftalovich R. Oydanich M. Iskander A. J. Naftalovich D. (2021). A sudden presentation of abdominal compartment syndrome. Anaesthesiol. Intensive Ther. 53 (1), 93–96. 10.5114/ait.2021.103513 33586415 PMC10158450

[B4] Bu L. Yang D. Nie F. Li Q. Wang Y. F. (2020). Correlation of the type and degree of cystocele with stress urinary incontinence by transperineal ultrasound. J. Med. Ultrason. 47 (1), 123–130. 10.1007/s10396-019-00972-0 31493276

[B5] Cameron A. P. (2019). Systematic review of lower urinary tract symptoms occurring with pelvic organ prolapse. Arab. J. Urol. 17 (1), 23–29. 10.1080/2090598X.2019.1589929 33110659 PMC7567315

[B6] Cameron A. P. Clemens J. Q. Latini J. M. McSuire E. J. (2016). Combination drug therapy improves compliance of the neurogenic bladder. J. Urol. 182 (3), 1062–10627. 10.1016/j.juro.2009.05.038 19616807

[B7] Çelebi S. Kuzdan Ö. Özaydın S. Başdaş C. B. Özaydın İ. Erdoğan C. (2016). A bladder diverticulum model in rabbits. J. Pediatr. Urol. 12 (5), 311.e1–311.e6. 10.1016/j.jpurol.2016.03.010 27139999

[B8] Dörflinger A. Monga A. (2001). Voiding dysfunction. Curr. Opin. Obstet. Gynecol. 13 (5), 507–512. 10.1097/00001703-200110000-00010 11547032

[B9] Habteyes F. G. Komari S. O. Nagle A. S. Klausner A. P. Heise R. L. Ratz P. H. (2017). Modeling the influence of acute changes in bladder elasticity on pressure and wall tension during filling. J. Mech. Behav. Biomed. Mater. 71, 192–200. 10.1016/j.jmbbm.2017.02.020 28343086 PMC5472102

[B10] Harris R. L. Cundiff G. W. Theofrastous J. P. Bump R. C. (1996). Bladder compliance in neurologically intact women. Neurourol. Urodyn. 15 (5), 483–488. 10.1002/(SICI)1520-6777(1996)15:5<483::AID-NAU5>3.0.CO;2-B 8857616

[B11] Hsieh M. F. Tsai H. W. Liou W. S. Lo C. C. Lin Z. H. An Y. F. (2019). Long-term compliance of vaginal pessaries: does stress urinary incontinence matter? Med. Baltim. 98 (14), e15063. 10.1097/MD.0000000000015063 30946355 PMC6455947

[B12] Iglesia C. B. Smithling K. R. (2017). Pelvic organ prolapse. Am. Fam. Physician 96 (3), 179–185. 28762694

[B13] Kummeling M. T. M. Rietbergen J. B. W. Withagen M. I. J. Mannaerts G. H. H. van der Weiden R. M. F. (2013). Sequential urodynamic assessment before and after laparoscopic sacrocolpopexy. Acta Obstet. Gynecol. Scand. 92 (2), 172–177. 10.1111/aogs.12045 23157606

[B14] Lau H. H. Lai C. H. Peng H. Y. Hsieh M. C. Su T. H. Lee J. J. (2022). Modification of bladder thermodynamics in stress urinary incontinence patients submitted to trans-obturator tape: a retrospective study based on urodynamic assessment. Front. Bioeng. Biotechnol. 10, 912602. 10.3389/fbioe.2022.912602 36061421 PMC9437260

[B15] Lau H. H. Lai C. H. Hsieh M. C. Peng H. Y. Chou D. Su T. H. (2023). Pressure-volume loop analysis of voiding workload-An application in trans-vaginal mesh repaired pelvic organ prolapse patients. Bioeng. (Basel) 19 (7), 853. 10.3390/bioengineering10070853 37508880 PMC10376103

[B16] Lau H. H. Lai C. H. Hsieh M. C. Peng H. Y. Chou D. Su T. H. (2024a). Effect of intra-vaginal electric stimulation on bladder compliance of stress urinary incontinence patients: the involvement of autonomic tone frontier neuroscience. Front. Neurosci. 18, 1432616. 10.3389/fnins.2024.1432616 39170685 PMC11337866

[B17] Lau H. H. Lai C. H. Hsieh M. C. Peng H. Y. Chou D. Su T. H. (2024b). Thermodynamic work of high-grade uterine prolapse patients undergo transvaginal mesh repair with total hysterectomy. Bioeng. (Basel) 28 (9), 875. 10.3390/bioengineering11090875 PMC11428840 39329617

[B18] Lau H. H. Su T. H. Lee J. J. Chou D. Hsieh M. C. Lai C. H. (2024c). Bladder compliance dynamics of pelvic organ prolapse in women undergoing robotic-assisted sacrocolpopexy. J. Minim. Invasive Gynecol. 31 (12), 1034–1040. 10.1016/j.jmig.2024.08.017 39233274

[B19] Lau H. H. Su T. H. Lee J. J. Chou D. Hsieh M. C. Lai C. Y. (2024d). Pressure-volume analysis of thermodynamic workload of voiding - an application in pelvic organ prolapse patients subjected to robotic-assisted sacrocolpopexy. Biomed. Eng. Lett. 15 (2), 357–365. 10.1007/s13534-024-00453-5 40026886 PMC11871214

[B20] Long C. Y. Hsu S. C. Sun D. J. Chen C. C. Tsai E. M. Su J. H. (2002). Abnormal clinical and urodynamic findings in women with severe genitourinary prolapse. Kaohsiung J. Med. Sci. 18 (12), 593–597. 12670034

[B21] Long C. Y. Hsu C. S. Wu C. H. Liu C. M. Wang C. L. Tsai E. M. (2012). Three-year outcome of transvaginal mesh repair for the treatment of pelvic organ prolapse. Eur. J. Obstet. Gynecol. Reprod. Biol. 161, 105–108. 10.1016/j.ejogrb.2011.12.007 22226537

[B22] Loo Z. X. Chen H. S. Tang F. H. Lin K. L. Liu Y. Y. Wu M. P. (2020). Predictors of voiding dysfunction following uphold™ mesh repair for the treatment of pelvic organ prolapse. Eur. J. Obstet. Gyneco.l Reprod. Biol. 255, 34–39. 10.1016/j.ejogrb.2020.09.041 33070088

[B23] Luo D. Y. Yang T. X. Shen H. (2018). Long term Follow-up of transvaginal anatomical implant of mesh in pelvic organ prolapse. Sci. Rep. 8 (1), 2829. 10.1038/s41598-018-21090-w 29434209 PMC5809369

[B24] Lyu L. Yao Y. X. Liu E. P. Zhang Y. P. Hu H. J. Ji F. P. (2022). A study of urodynamic parameters at different bladder filling stages for predicting upper urinary tract dilatation. Int. Neurourol. J. 26 (1), 52–59. 10.5213/inj.2142244.122 35368186 PMC8984689

[B25] Ma Y. Kang J. Zhang Y. Ma C. Wang Y. Zhu L. (2021). Medium-term effects on voiding function after pelvic reconstructive surgery of advanced pelvic organ prolapse: is postoperative uroflowmetry necessary? Eur. J. Obstet. Gynecol. Reprod. Biol. 258, 447–451. 10.1016/j.ejogrb.2020.09.038 33082050

[B26] Majkusiak W. Horosz E. Tomasik P. Zwierzchowska A. Wielgoś M. Barcz E. (2015). Quality of life assessment in women after cervicosacropexy with polypropylene mesh for pelvic organ prolapse: a preliminary study. Menopausal Rev. 14 (2), 126–129. 10.5114/pm.2015.52153 26327900 PMC4498030

[B27] Martini N. Hanna M. Alshwaiki A. Aldeen B. A. (2022). Inguinal bladder hernia (IBH) managed by lichtenstein technique: a case report. Int. J. Surg. Case Rep. 99, 107617. 10.1016/j.ijscr.2022.107617 36152366 PMC9568717

[B28] Martini N. Kara T. N. Aldarwish M. S. Mahmoud J. (2023). Rectal prolapse as a manifestation of inflammatory bowel disease with celiac disease in a 2-year-old male: a rare case report. Ann. Med. Surg. 85 (4), 1235–1239. 10.1097/MS9.0000000000000494 37113926 PMC10129242

[B29] Mateu-Arrom L. Gutiérrez-Ruiz C. Palou R. J. Errando-Smet C. (2021). Pelvic organ prolapse repair with mesh: description of surgical technique using the Surelift®Anterior repair system. Urol. Int. 105, 137–142. 10.1159/000510530 33075779

[B30] Mills I. W. Drake M. J. Greenland J. E. Nobel J. G. Brading A. F. (2000). The contribution of cholinergic detrusor excitation in a pig model of bladder hypocompliance. BJU Int. 86 (4), 538–543. 10.1046/j.1464-410x.2000.00768.x 10971288

[B31] Naumann G. Hüsch T. Mörgeli C. Kolterer A. Tunn R. (2021). Mesh-augmented transvaginal repair of recurrent or complex anterior pelvic organ prolapse in accordance with the SCENIHR opinion. Int. Urogynecol. J. 32, 819–827. 10.1007/s00192-020-04525-9 32970175 PMC8009781

[B39] Peng H. Y. Lai C. Y. Hsieh M. C. Ho Y. C. Lin T. B. (2012). Pressure-volume analysis of rat’s micturition cycles *in vivo* . Neurourol. Urodyn. 39 (5), 1304–1312. 10.1002/nau.24363 32293055 PMC7318613

[B32] Peng H. Y. Lai C. Y. Hsieh M. C. Lin T. B. (2021). Solifenacin/mirabegron induces an acute compliance increase in the filling phase of the capacity-reduced urinary bladder: a pressure-volume analysis in rats. Front. Pharmacol. 12, 657959. 10.3389/fphar.2021.657959 34122078 PMC8188241

[B33] Rogowski A. Kluz T. Szafarowska M. Mierzejewski P. Sienkiewicz-Jarosz H. Samochowiec J. (2019). Efficacy and safety of the calistar and elevate anterior vaginal mesh procedures. Eur. J. Obstet. Gynecol. Reprod. Biol. 239, 30–34. 10.1016/j.ejogrb.2019.05.033 31163354

[B34] Sugaya K. Nishizawa O. Satoh T. Hatano T. Ogawa Y. (2000). Bladder-pumping therapy for the treatment of low-capacity or low-compliance bladders. Neurourol. Urodyn. 19 (1), 19–28. 10.1002/(sici)1520-6777(2000)19:1<19::aid-nau4>3.0.co;2-3 10602245

[B35] Toppercer A. Tetreault J. P. (1979). Compliance of the bladder: an attempt to establish normal values. Urology 14 (2), 204–205. 10.1016/0090-4295(79)90164-x 473478

[B36] US Food and Drug Administration (2016). FDA news release: FDA strengthens requirement for surgical mesh for the transvaginal repair of pelvic organ prolapse to address safety risks. Silver Springs., MD U. S. Food Drug Adm.

[B37] Wu C. Q. Franco I. (2017). Management of vesicoureteral reflux in neurogenic bladder *investig* . Clin. Urol. 58 (Suppl. 1), S54–S58. 10.4111/icu.2017.58.S1.S54 28612061 PMC5468266

[B38] Yokoyama O. Mita E. Ishiura Y. Nakamura Y. Nagano K. Namiki M. (1997). Bladder compliance in patients with benign prostatic hyperplasia. Neurourol. Urodyn. 16 (1), 19–27. 10.1002/(sici)1520-6777(1997)16:1<19::aid-nau2>3.0.co;2-h 9021787

