# Supplementary figures and images for: Uneven effects of trans-vaginal mesh reconstruction on the viscoelastic property of the urinary bladder in patients with pelvic organ prolapse
Source: Front Bioeng Biotechnol. 2026 Jan 12;13:1677779. doi: 10.3389/fbioe.2025.1677779 (PMC12832731; doi:10.3389/fbioe.2025.1677779)

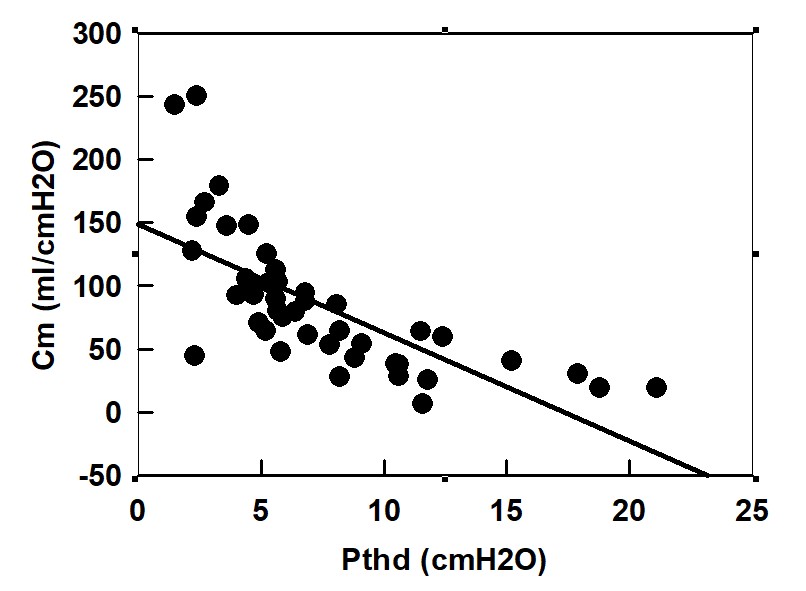

Supplement: Supplementary file 2 [file Image3.jpeg]

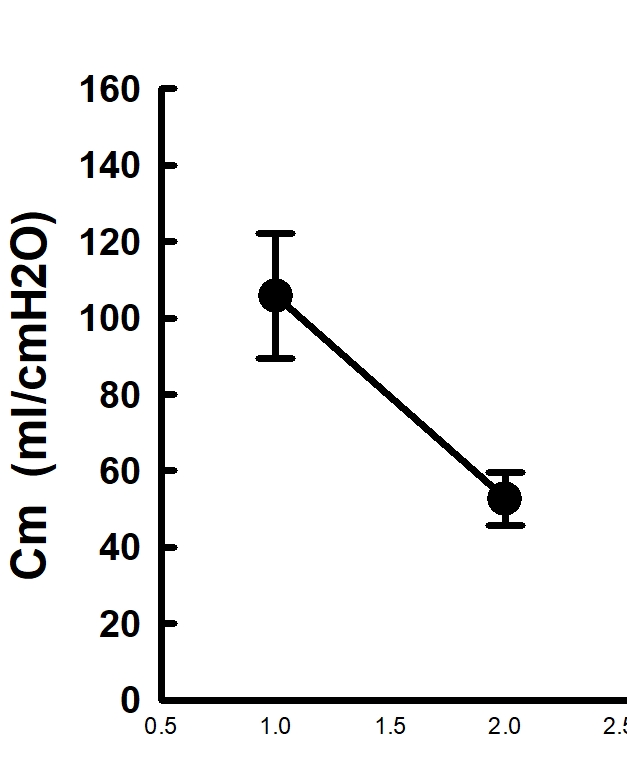

Supplement: Supplementary file 4 [file Image9.jpeg]

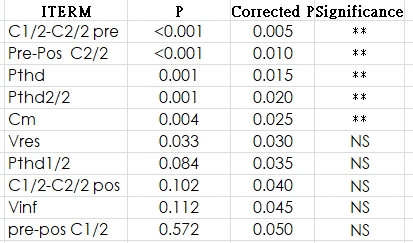

Supplement: Supplementary file 5 [file Image1.jpeg]

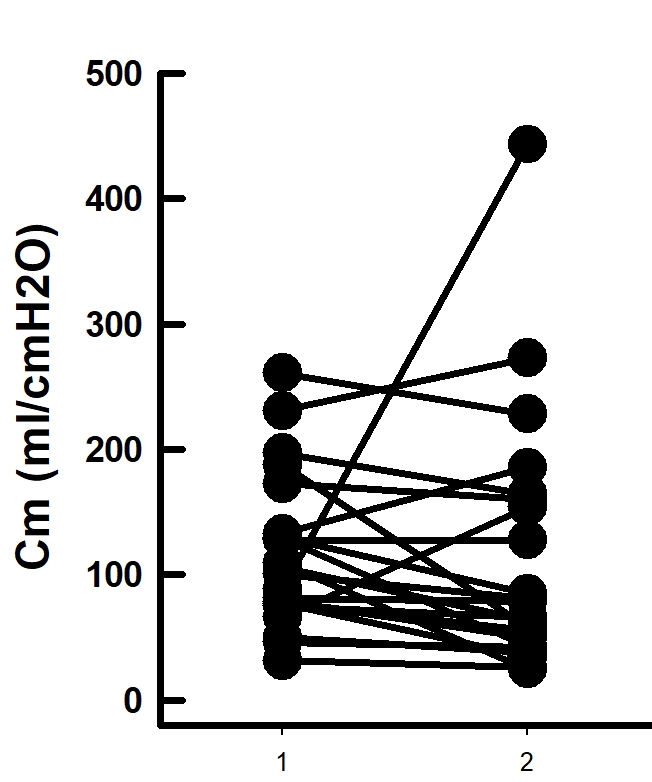

Supplement: Supplementary file 6 [file Image4.jpeg]

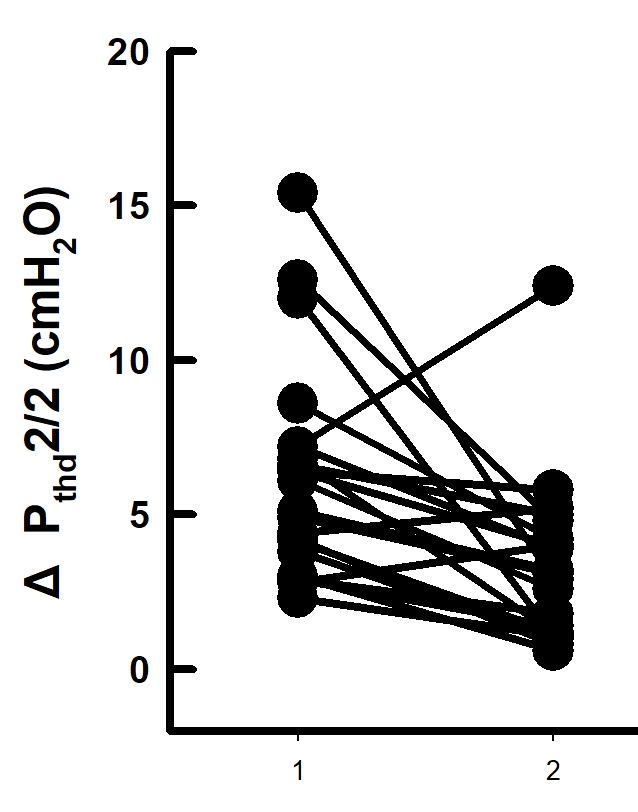

Supplement: Supplementary file 7 [file Image7.jpeg]

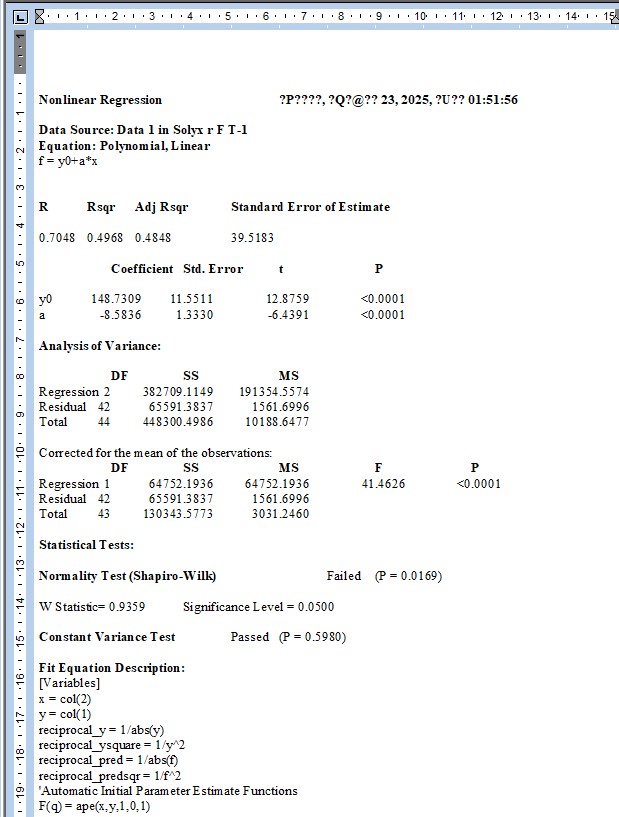

Supplement: Supplementary file 8 [file Image2.jpeg]

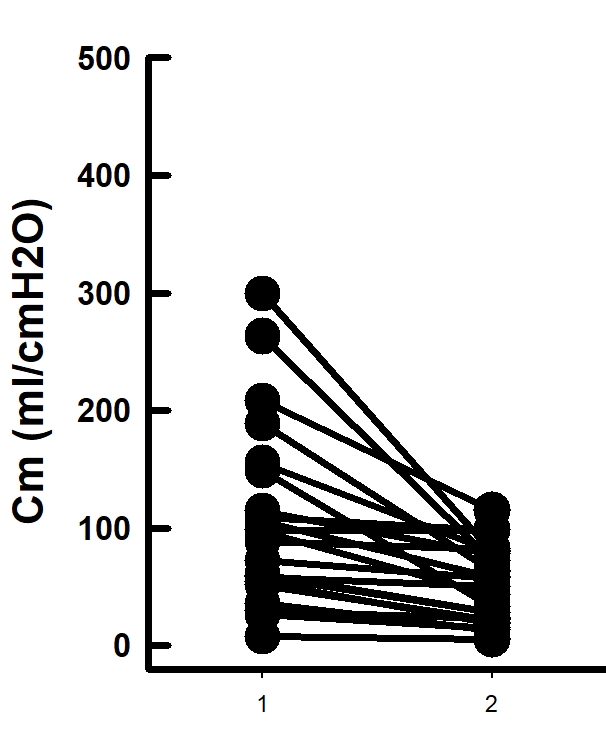

Supplement: Supplementary file 9 [file Image5.jpeg]

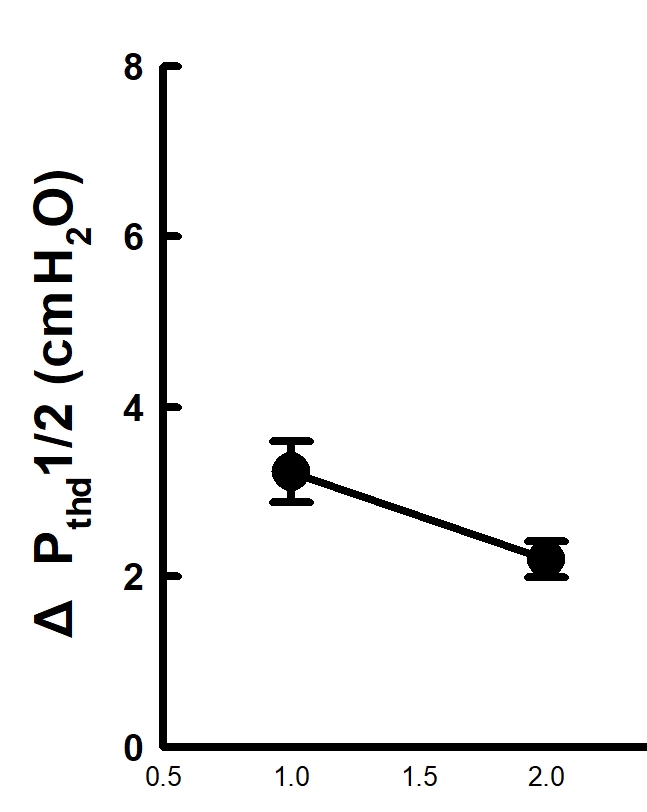

Supplement: Supplementary file 10 [file Image10.jpeg]

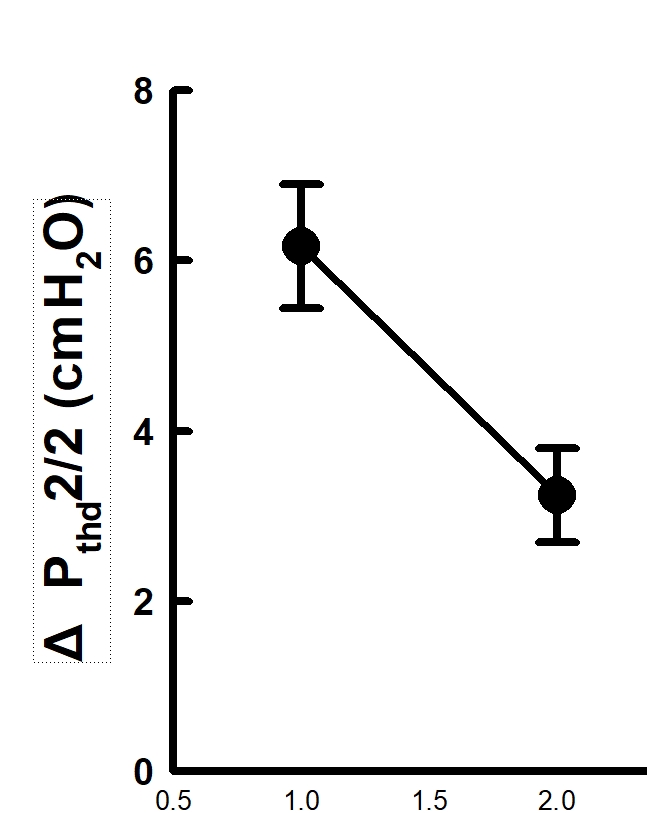

Supplement: Supplementary file 11 [file Image11.jpeg]

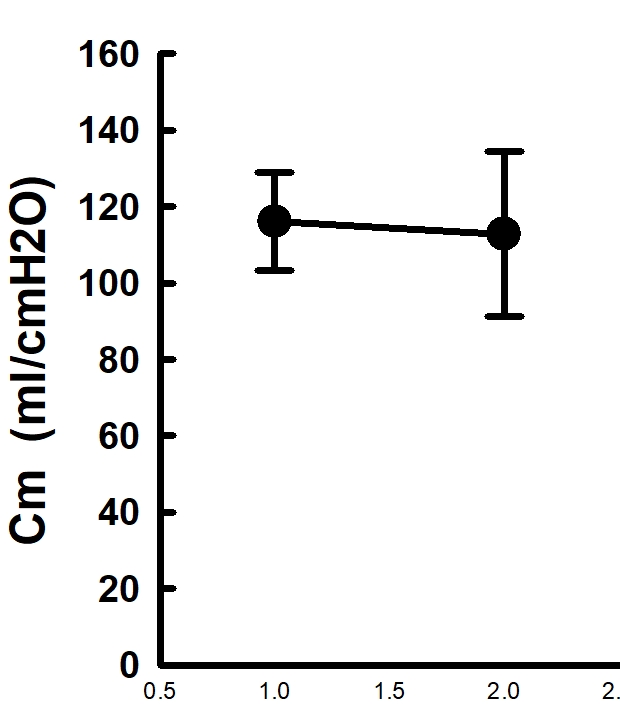

Supplement: Supplementary file 13 [file Image8.jpeg]

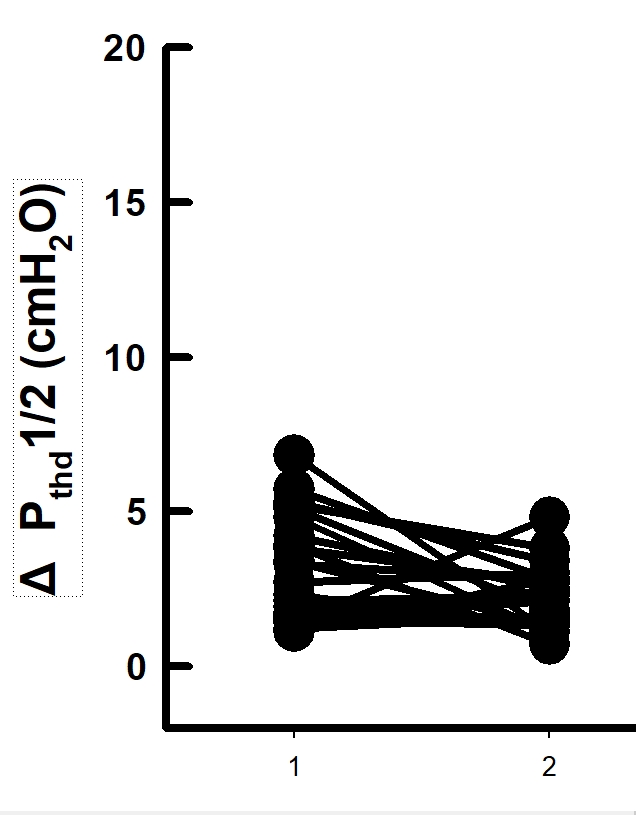

Supplement: Supplementary file 14 [file Image6.jpeg]
